# Immunomodulatory activity of aqueous extract from *Crassostrea sikamea* in the splenocytes of Sprague‐Dawley rats

**DOI:** 10.1002/fsn3.2710

**Published:** 2022-01-05

**Authors:** Guannan Guo, Ying Kong, Jie Su, Geng Wang, Muqing Zhang, Shuyue Wang, Zhenbo Song

**Affiliations:** ^1^ National Engineering Laboratory for Druggable Gene and Protein Screening School of Life Sciences Northeast Normal University Changchun China; ^2^ NMPA Key Laboratory for Quality Control of Cell and Gene Therapy Medicine Products Northeast Normal University Changchun China; ^3^ School of Molecular & Cellular Biology University of Illinois Urbana Champaign Urbana Illinois USA

**Keywords:** aqueous extract, *Crassostrea sikamea*, immunomodulatory activity, splenocyte

## Abstract

*Crassostrea sikamea* (*C*. *sikamea*) is used as an important edible and medicinal seafood in China. In the present study, an aqueous extract of *C*. *sikamea* (AECs) was prepared, and its immunomodulatory effects on rat splenocytes were studied. 3‐(4,5‐dimethylthiazol‐2‐yl)‐5‐(3‐carboxymethoxyphenyl)‐2‐(4‐sulfophenyl)‐2H‐tetrazolium (MTS) assay revealed that AECs was able to promote splenocyte proliferation. Moreover, flow cytometry revealed that AECs treatment markedly altered the populations of splenic lymphocyte subtypes. Data from real‐time quantitative PCR (RT‐qPCR) and enzyme‐linked immunosorbent assay (ELISA) showed that AECs promoted the mRNA expression and secretion of TNF‐*α*, IL‐2, IL‐6, IL‐12, and IFN‐γ. Mechanistically, p38 MAPK phosphorylation in splenocytes was significantly upregulated under AECs treatment and p38 MAPK inhibitor reversed the promoting effect of AECs on the expression of inflammatory cytokines. Collectively, our novel evidence suggests that AECs exhibits immunomodulatory activity in vitro, supporting the further application of *C*. *sikamea* as a potential functional food.

## Graphical abstract

AECs promotes the proliferation of splenocytes, regulates lymphocyte subsets, and alters the expression and secretion of inflammatory cytokines in vitro.

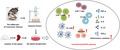

## 1 INTRODUCTION

*Crassostrea sikamea* oyster is one of the species of *Crassostrea* belonging to the Ostreidae family, which includes a number of economically important mariculture species that are naturally distributed in China (Wang et al., 2014) and are abundant over a wide geographical area worldwide. Traditionally, oysters are referred to as “the milk of the sea” because they are an excellent source of high‐quality nutrition, including polysaccharides, protein, lipids, peptides, phenolic compounds, and minerals. (Kit‐Leong et al., 2017; Wang, Li, He, et al., 2014). In addition to their nutritional value, oysters have considerable potential in the functional food industry, exhibiting beneficial health effects. Previous studies have demonstrated that oyster extracts possessed numerous biological properties, such as antitumor (Sakaguchi et al., 2018), antimicrobial (Huang et al., 2019; Karthikeyan et al., 2014), antioxidant (Watanabe et al., 2012), and immunomodulatory effects (Cheng et al., 2013; Li et al., 2019).

*Crassostrea sikamea* oyster is known for its smooth texture and sweet fruity flavor despite its small size and slow growth. This species has been cultivated and represents a locally important fishery resource in China, Japan, Korea, the United States, and some European countries (Xu, Li et al., 2019; Yu & Li, 2012). However, only a few studies on the chemical constituents and biological activity of *C*. *sikamea* oysters have been reported. Furthermore, the immunomodulatory activity of *C*. *sikamea* and the mechanism involved in the immunomodulatory effects have not been investigated.

To assess the immunomodulatory effects of *C*. *sikamea*, we collected the AECs and investigated its immunomodulatory activity in the splenocytes of *SD* rats in this study. The current results revealed that AECs promoted the proliferation of splenocytes and altered the proportion of splenic lymphocyte subtypes in vitro. Furthermore, we found that AECs regulated the secretion of inflammatory cytokines in splenocytes. Our present study provided novel evidence suggesting that *C*. *sikamea* exhibited considerable potential in immunoregulation, which could be helpful in further understanding the biological activity of the *Ostreidae* family.

## 2 MATERIALS AND METHODS

### 2.1 Preparation of AECs

*Crassostrea sikamea* material was obtained from the city of Zhanjiang (Guangdong Province, China) and transported to our laboratory. The edible parts were collected and samples were subsequently freeze‐dried with liquid nitrogen and milled into powders. Sample powders (5.0 g) were extracted with 150 ml of acetone (*v*/*v*) using an ultrasound processer (480 W, 24 KHZ, Kangshijie Ultrasonic Wave Tech., Dongguan, China) at 60°C for 30 min. Then, centrifugation at 4000 *g* was performed for 20 min. The residues were collected for further extraction of AECs. Briefly, the residues were refluxed twice with 150 ml water at 95°C for 2 hr. After centrifugation (4500 *g* for 20 min), the supernatant was combined and collected. The combined extracts were concentrated under vacuum at 60°C. Three volumes of 95% (*v*/*v*) ethanol were used for the precipitation of the AECs in the supernatant at 4°C overnight. Trace proteins were eliminated using Savage method. Then, the crude extract was collected after lyophilization by a vacuum freeze drier (Lyo Quest, Telstar) for 8 h and referred to as AECs.

### 2.2 Animals

Male Sprague‐Dawley rats (8–10 weeks) were purchased from the Experimental Animal Center, Jilin University (Changchun, Jilin, China). Rats were housed in a temperature‐ and humidity‐controlled environment with a 12‐h/12‐h light/dark cycle. Rats were given free access to food and water in the cage during the experiment. All animal experiments were performed in accordance with protocols approved by the Ethics Committee of Northeast Normal University (NENU/IACUC, AP 20,171,023).

### 2.3 Preparation and culture of rat splenocytes

The spleens of the rats were minced in D‐Hanks medium (Solarbio, Beijing, China) and pressed through a 100‐μm fine wire mesh screen. Cell mixtures were collected in 15‐ml centrifugal tubes and centrifuged at 400 *g* for 10 min at 4°C. After removing the supernatant, the cell pellet was acquired, and 1 ml of lysis buffer (Solarbio, Beijing, China) was added to remove red blood cells. Then, the cells were washed thrice with cold D‐Hanks medium. Subsequently, the cells were adjusted to a concentration of 1 × 10^6^ cells/ml in RPMI‐1640 medium (Gibco, CA, USA) with 10% (V/V) fetal bovine serum (Gibco, CA, USA), 100 U/ml penicillin and 100 μg/ml streptomycin. All cells were incubated in a standard incubator at 37°C with 5% CO_2_.

### 2.4 Cell viability assay

The MTS assay (Promega, WI, USA) was used to evaluate the viability of splenocytes according to the manufacturer's instructions. The cells were separately incubated with AECs at different concentrations (0, 5, 25, and 100 μg/ml) and concanavalin A (ConA, 5 μg/ml) for 48 h in a 96‐well plate. ConA was used as positive control. After incubation for 48 h, MTS was added to each well and incubated for another 3 h. The optical density (OD) values were measured at 490 nm using an automated microplate reader (Molecular Devices, LLC).

### 2.5 Flow cytometric analysis

Splenocytes were treated with AECs for 48 h, collected in a 1.5‐ml centrifuge tube and then stained with primary antibodies, including anti‐rat CD3‐FITC, CD4‐PE, CD8‐APC, CD45‐RA+, CD161‐PE, CD80‐PE, CD86‐FITC, and CD103‐Alexa Flox@647 (eBiosciences, CA, USA). As reported, we defined the CD3^+^CD4^+^ population as helper T (Th) cells, CD3^+^CD8^+^ population as cytotoxic T (Tc) cells, CD3^‐^CD45RA^+^ population as B lymphocytes, CD3^‐^CD161^+^ population as natural killer (NK) cells, CD3^+^CD161^+^ population as natural killer T (NKT) cells, and CD80‐PE, CD86‐FITC, and CD103‐Alexa Flox@647 as dendritic (DC) cells (Ayako et al., 2018; Chen et al., 2018; Xu, Wusiman, et al., 2019). After incubation at 4°C for 30 min, the cells were washed twice and resuspended in PBS before they were transferred to fluorescence‐activated cell sorting (FACS) tubes and analyzed by flow cytometry (BD Biosciences, San Jose, CA, USA). The cells were gated using forward and side scatter for dead cell exclusion. In each sample, 10,000 events were measured, and data were analyzed using Flow Jo 7.6 software.

### 2.6 RNA isolation and real‐time PCR

Total RNA was extracted from splenocytes using TRIzol reagent (Invitrogen, CA, USA) and then reverse transcribed using TransScript SuperMix (TransGen Biotech, Beijing, China) according to the manufacturer's instructions. The mRNA levels of TNF‐*α*, IL‐2, IL‐6, IL‐12, and IFN‐γ were evaluated by real‐time PCR using FastStart Universal SYBR Green Master Mix (Roche, Mannheim, Germany) and a Bio‐Rad real‐time PCR detection system. All primers from 5′ to 3′ end were listed as follows: TNF‐*α*, F: GCCTCCTCTCTGCCATCAAG and R: CTCCAAAGTAGACCTGCCCG; IL‐6, F: TCTGCTCTGGTCTTCTGGAGT and R: GCATTGG AAGTTGGGGTAGGA; IL‐12, F: AGTTCTTCGTCCGCATCCAG and R: CTTGCACGCAGAT ATTCGCC; IL‐2, F: CCCTGCAAAGGAAACACAGC and R: CAAATCCAACACACGCTGCA; IFN‐γ, F: CAACCCACAGATCCAGCACA and R: TCAGCACCGACTCCTTTTCC; GAPDH, F: GACATGCCGCCTGGAGA AAC and R: AGCCCAGGATGCCCTTTAGT.

### 2.7 Cytokine production assay

Splenocytes were seeded in six‐well plates and incubated with AECs at 100 μg/ml for 48 h. The TNF‐*α*, IL‐2, IL‐6, IL‐12, and IFN‐γ levels in the supernatant were measured by ELISA kits (Shanghai Enzyme‐linked Biotechnology Co., Ltd, Shanghai, China) using the quantitative sandwich enzyme immunoassay technique according to the manufacturer's instructions.

### 2.8 Western blotting

Whole cell lysates from splenocytes were prepared and subjected to Western blotting as previously described (Wang et al., 2019). Antibodies against p38, p‐p38, JNK, and p‐JNK were purchased from Cell Signaling Technology (Cell Signaling Technology, Beverly, MA, USA). Antibodies against ERK, p‐ERK, and GAPDH were provided by Proteintech (Proteintech, Wuhan, China).

### 2.9 Statistical analysis

Data were presented as mean ± *SD* based on three independent experiments. All results were analyzed by Graphpad Prism 9 software (GraphPad Software, San Diego, CA, USA). The significance of the differences between two groups was analyzed by Student's *t*‐test. Multiple group comparisons were analyzed by one‐way ANOVA. In all analysis, a *p*‐value < 0.05 was regarded as statistically significant.

## 3 RESULTS

### 3.1 AECs treatment promotes the proliferation of splenocytes

**FIGURE 1:**
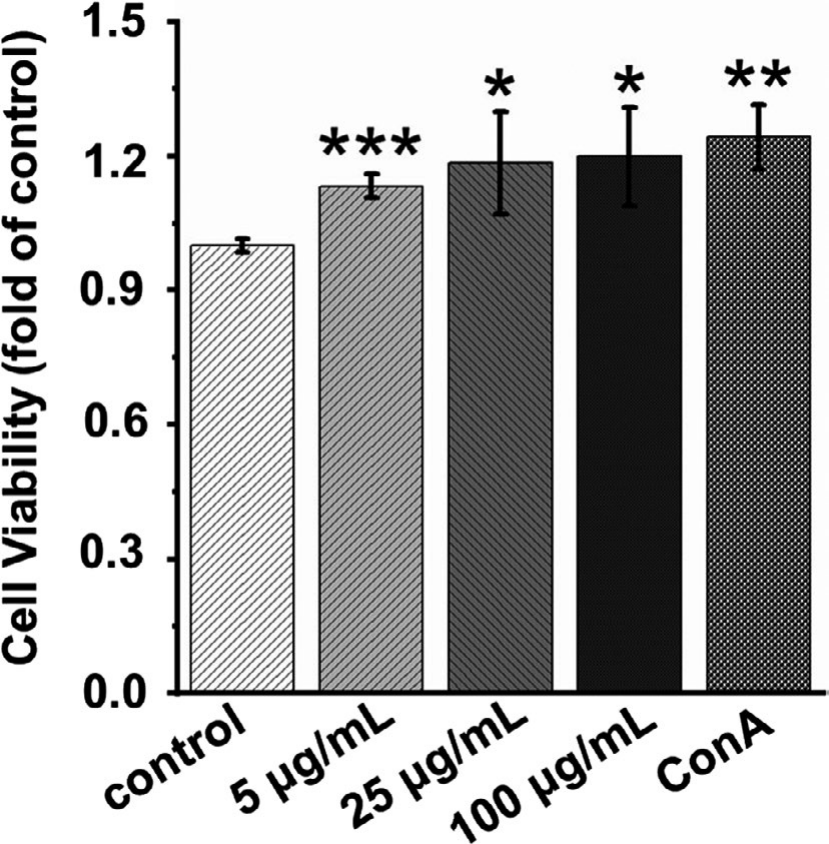
Effect of AECs on the viability of splenic lymphocyte of *SD* rats. Splenic lymphocytes were treated with different concentrations of AECs (5, 25, and 100 μg/ml), RPMI‐1640 medium (as a negative control), or concanavalin A (ConA, 5 μg/ml, as a positive control). After 48 h of incubation, cell viability was evaluated by the MTS assay. Data are expressed as the mean ± *SD* (*n* = 3). *p < .05, **p < .01 and ***p < .001

Splenocyte proliferation is a direct indicator of cellular immunity (Kit‐Leong et al., 2017). To assess whether AECs exerts an immunomodulatory effect in vitro, the effects of different concentrations of AECs on splenocyte viability in *SD* rats were investigated. As shown in Figure 1, treatment of splenocytes with 5, 25, and 100 µg/ml AECs for 48 h significantly promoted splenocyte viability in a dose‐dependent manner compared to that of untreated splenocytes. Based on these results, a concentration of 100 µg/ml ACEs was selected for subsequent experiments.

### 3.2 AECs treatment alters the populations of splenic lymphocyte subtypes

To further investigate the effects of AECs on the percentages of splenic lymphocyte subtypes in *SD* rats, we performed a thorough immunophenotyping of the different cell subsets present in the spleens using flow cytometric analysis.

**FIGURE 2:**
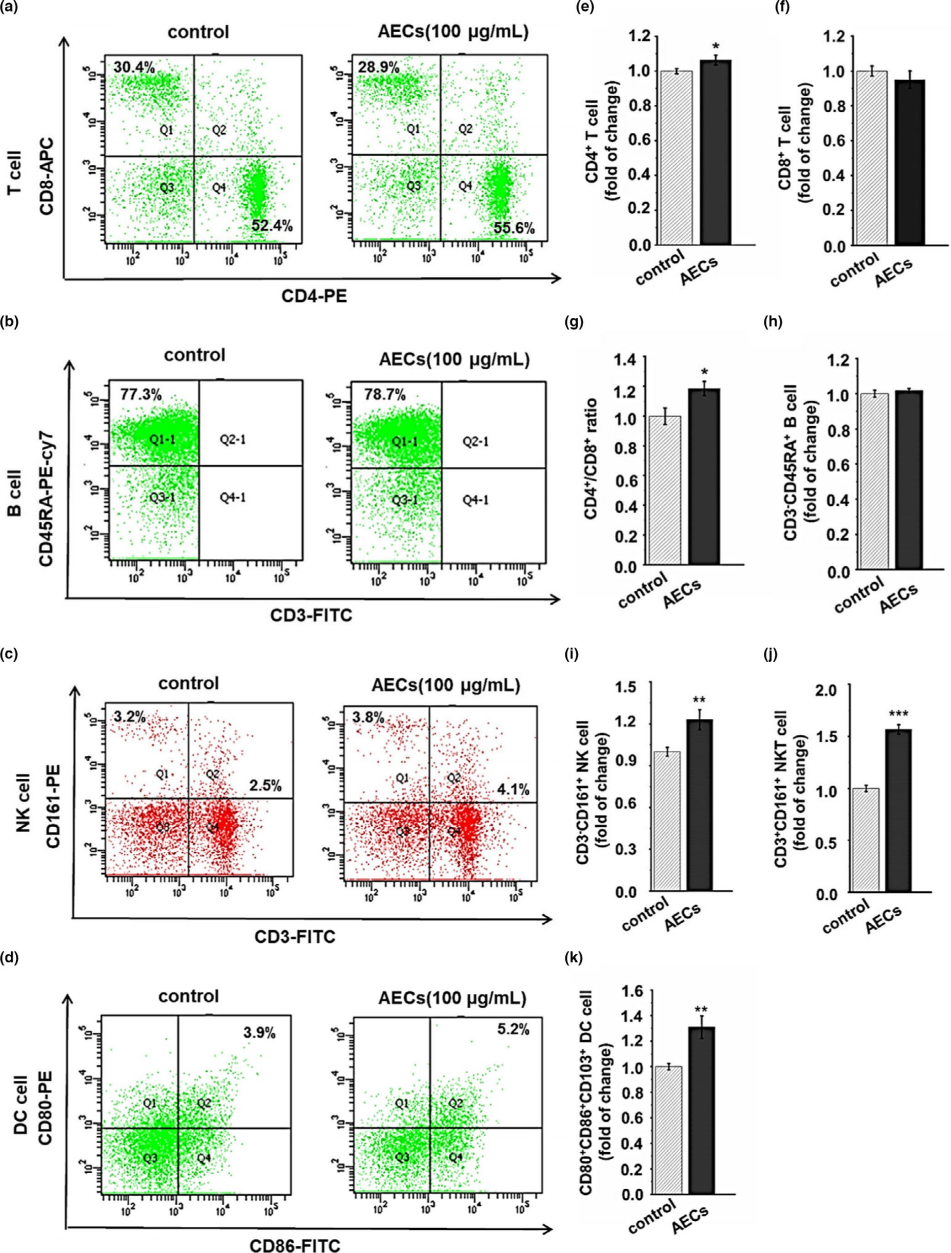
Effects of AECs on the proportion of splenic lymphocyte subtypes. The proportions of T cells, B cells, NK cells, NKT cells, and DC cells were detected by specific antibodies and evaluated by flow cytometry (a–d). Their quantitations are shown in Figure 2e–k. The results are expressed as the mean ± *SD* (*n* = 3). *p < .05, **p < .01, ***p < .001

As illustrated in Figure 2(a,e–g), we conducted a phenotypic analysis of the total T cells and the T cell subsets. The percentages of CD3^+^CD4^+^ T lymphocytes were increased under AECs treatment. However, no significant changes in the percentages of CD3^+^CD8^+^ T lymphocytes were noted. Moreover, we found that the CD3^+^CD4^+^/CD3^+^CD8^+^ ratio of the AECs‐treated group was 11.82% greater than that of the control group. As shown in Figure 2(b,h)), there were no significant changes in the percentages of CD3^‐^CD45RA^+^ B lymphocytes between the AECs and control group. We also observed that AECs treatment significantly increased the proportion of CD3^‐^CD161^+^ natural killer cells (NK cells), CD3^+^CD161^+^ natural killer T cells (NKT cells), and CD80^+^CD86^+^CD103^+^ dendritic cells (DC cells) by 22.92% (Figure 2c,i), 56.58% (Figure 2c,j), and 30.83% (Figure 2d,k).

### 3.3 AECs treatment regulates the mRNA expression and secretion of inflammatory cytokines in splenocytes

**FIGURE 3:**
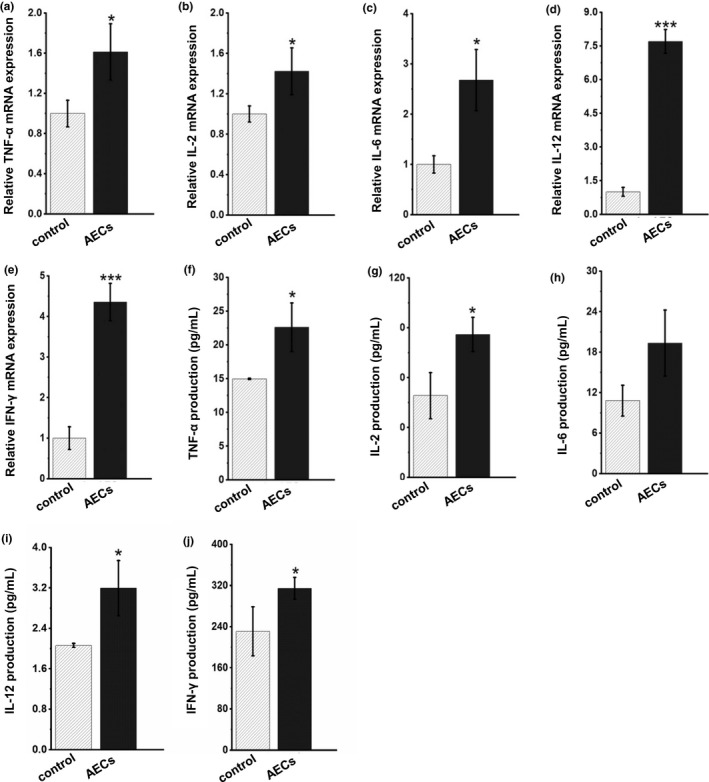
Effects of AECs on the expression and secretion of inflammatory cytokines in splenic lymphocytes. Splenocytes were treated with AECs (100 μg/ml), and the expression and secretion levels of the cytokines TNF‐*α* (a, f), IL‐2 (b, g), IL‐6 (c, h), IL‐12 (d, i), and IFN‐γ (e, j) were determined by RT‐PCR analysis and ELISA. The data are presented as the mean ± *SD* (*n* = 3). *p < .05, ***p < .001 compared with the control group

Cytokines are critical regulators of the immune system that activate and modulate the function of immune cells (Charles & Dinarello, 2007). Activated splenocytes generate and sequentially secrete a variety of inflammatory cytokines, such as TNF‐*α*, IL‐2, IL‐6, IL‐12, and IFN‐γ (Zhang et al., 2017), which play important roles in the body's immune response. Thus, the mRNA and protein levels of inflammatory cytokines, including TNF‐*α*, IL‐2, IL‐6, IL‐12, and IFN‐γ, were measured by RT‐PCR and ELISA to determine the potential effects of AECs. As shown in Figure 3(a–j), AECs significantly increased the mRNA expression of TNF‐*α*, IL‐2, IL‐6, IL‐12, and IFN‐γ and secretion of TNF‐*α*, IL‐2, IL‐12, and IFN‐γ compared to the control group in splenocytes. These results indicated that AECs regulate the expression and secretion of inflammatory cytokines in splenocytes at both the transcriptional level and the protein level.

### 3.4 AECs treatment activates the p38 MAPK signaling pathway

**FIGURE 4:**
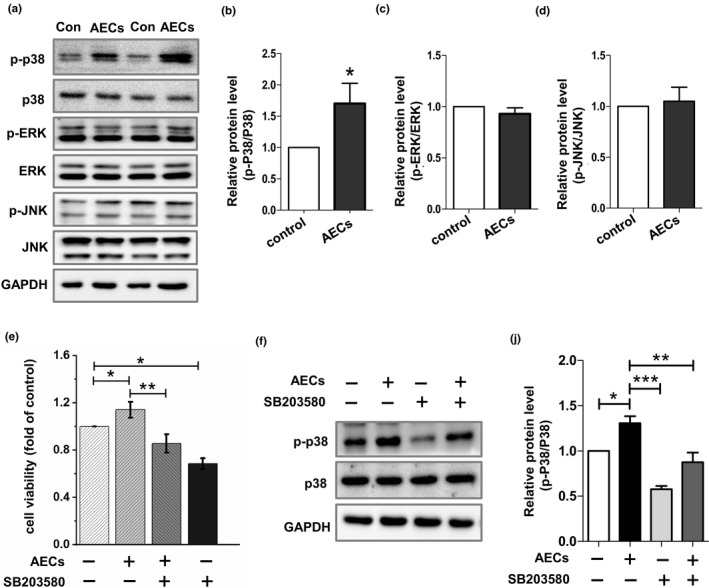
Effects of AECs and SB203580 on the phosphorylation of p38 and ERK and viability of splenic lymphocytes. Splenocytes were treated with AECs (100 μg/ml) for 24 h, and then treated with or without SB203580 (10 μM) for 3 h. The phosphorylation of p38 MAPK, ERK, and JNK was analyzed by western blotting (a–d, f, j). The cell viability was evaluated by the MTS assay (e). The data are presented as the mean ± *SD* (*n* = 3). *p < .05, **p < .01 compared with the control group

**FIGURE 5:**
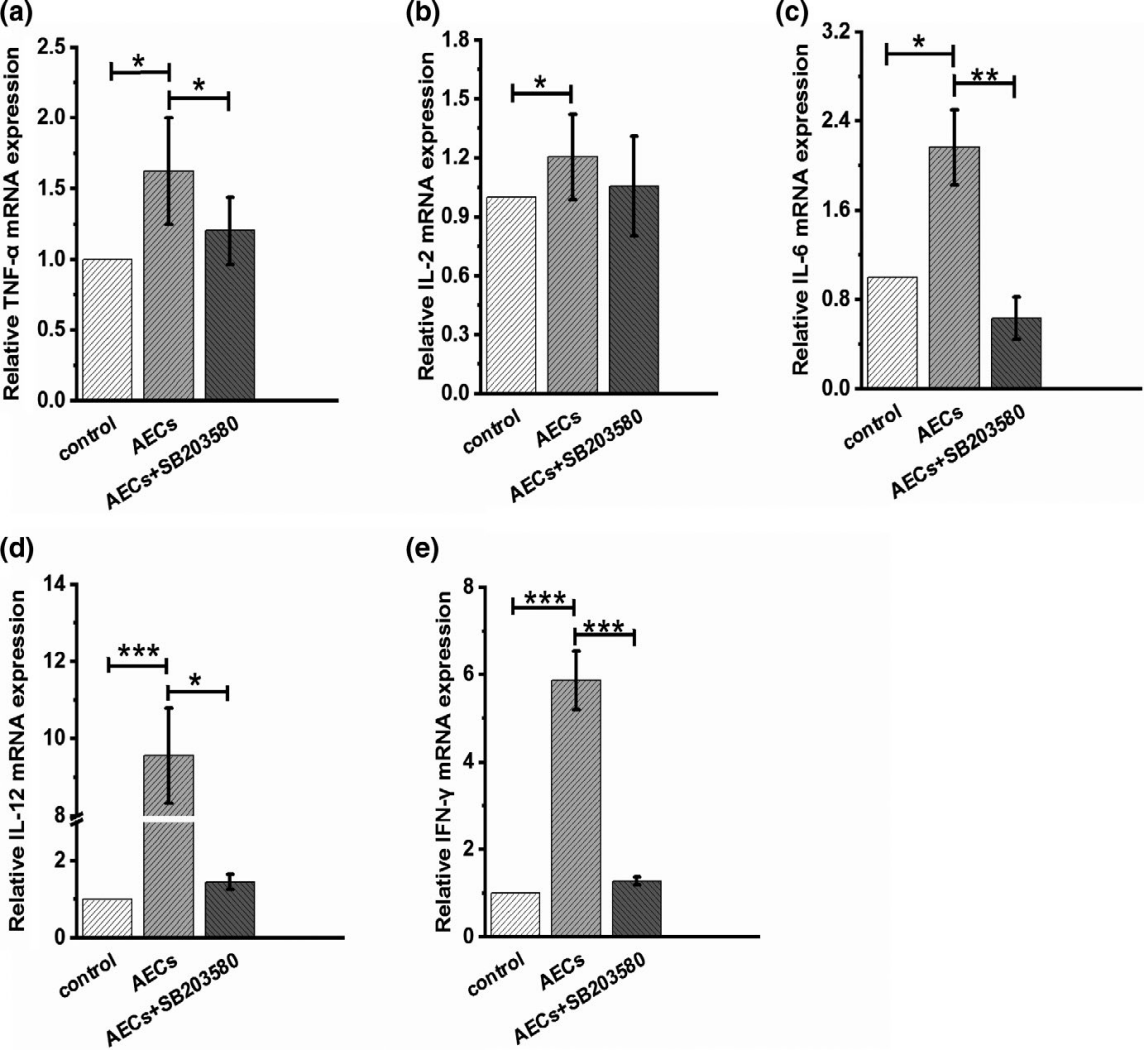
Effects of AECs and SB203580 on the mRNA expression of cytokines in splenic lymphocytes. Splenic lymphocytes were treated with AECs (100 μg/ml) for 24 h, and then treated with SB203580 (10 μM) for 3 h, and the expression of the cytokines TNF‐*α* (a), IL‐2 (b), IL‐6 (c), IL‐12 (d), and IFN‐γ (e) was determined by RT‐PCR analysis. The data are presented as the mean ± *SD* (*n* = 3). *p < .05, **p < .01, ***p < .001 compared with the control group

Next, to gain further insights into the underlying mechanism of AECs in immunoregulation, we used Western blotting to detect activation of the mitogen‐activated protein kinase (MAPK) signal transduction pathways. As reported, the MAPK signaling pathways are crucial in regulating immune responses and the expression of a variety of inflammatory cytokines (Simon et al., 2013; Tewari et al., 2015). Our results revealed that phosphorylated p38 MAPK levels in splenocytes were significantly increased when stimulated by AECs (Figure 4a,b). However, the phosphorylation levels of the extracellular signal‐regulated kinase 1/2 (ERK1/2) and the c‐Jun N‐terminal kinase/stress‐activated protein kinase (JNK) were not altered (Figure 4a‐d). Furthermore, we detected splenocyte proliferation under p38 MAPK pathway inhibitor SB203580 treatment. As shown in Figure 4(e), SB203580 significantly reduced the splenocyte viability and reversed the promoting effect of AECs on splenocyte proliferation. Western blotting result showed that SB203580 successfully inhibited the phosphorylation of p38 MAPK and AECs‐induced activation of p38 MAPK pathway was abolished by SB203580 (Figure 4f,j). In addition, we measured the mRNA levels of TNF‐*α*, IL‐2, IL‐6, IL‐12, and IFN‐γ in splenocytes when incubated with AECs and SB203580. Our results demonstrated that SB203580 reversed AECs‐induced upregulation of mRNA levels of TNF‐*α*, IL‐12, and IFN‐γ (Figure 5a–e). Thus, we speculate that AECs play an immunomodulatory role partly through p38 signal transduction pathway.

## 4 DISCUSSION

*Crassostrea sikamea* oyster is one of the most important marine mollusks and contributes greatly to the aquaculture industry. Oyster meat is an adequate nutritional resource with high functional value. Previous studies have reported that the constituents of oysters exhibit a variety of biological activities. For example, a water‐soluble polysaccharide isolated from *Crassostrea gigas* (CGPS‐1) possesses protective effects against acute carbon tetrachloride‐ and chronic ethanol‐induced liver injury in mice (Shi et al., 2015). Oligosaccharides and a phenolic antioxidant extracted from Crassostrea gigas exhibit strong antioxidant activities (Watanabe et al., 2012; Wu & Huang, 2017). Yin et al. found that oyster crude polysaccharides (OPS) could attenuate lipopolysaccharide (LPS)‐induced immune stress in weanling piglets (Yin et al., 2016). Moreover, another study demonstrated that treatment of human peripheral blood mononuclear cells (PBMCs) with an oyster extract enhanced IL‐2‐dependent T cell proliferation (Achour et al., 1997). The aim of our present study was to investigate the effects of the aqueous extracts of *C*. *sikamea* oysters on immunomodulatory activity in vitro.

Aqueous fractions are typically used in traditional Chinese medicine. First, we collected aqueous extracts from *C. sikamea* oysters (AECs). Moreover, we also obtained two crude extracts using two different extraction methods, namely, cold‐soaked extraction and percolation extraction, and six other crude extracts were extracted from different organic reagents. To identify potential immunomodulatory agents from *C. sikamea*, we treated the splenocytes of *SD* rats with these nine crude extracts, and the MTS results showed that AECs displayed more potent immunomodulatory activity (data not shown).

As an important organ of the immune system, the spleen plays a vital role in mediating the immune response and ensuring that a protective response to harmful stimuli is established (Springer, 1990). The proliferation of splenocytes is a direct indicator of cellular immunity. Our results showed that the in vitro exposure of rat splenocytes to AECs significantly enhanced cell proliferation suggesting that aqueous fraction of *C. sikamea* has the function of regulating cellular immunity. Similar to our present result, Cheng et al reported that an extract of oyster (*Crassostrea gigas*) polysaccharides (OPS) promoted the metabolic activity and proliferation of mouse splenocytes (Cheng et al., 2013).

Splenocytes are a mixture of various immune cells, such as T cells, B cells, DCs, and NK cells (Wynn et al., 2013). Moreover, lymphocyte proliferation and apoptosis affect the maintenance of the number and ratio of various lymphocyte subsets as well as the general function of immunoregulation. Our results showed that the percentage of CD3^+^CD4^+^ T lymphocytes and the ratio of CD3^+^CD4^+^/CD3^+^CD8^+^ T lymphocytes were increased under AECs treatment for 48 h. Studies have shown that activated T‐cell subpopulations can secrete a variety of immunologically active cytokines (Harris et al., 2000). The appropriate ratio and counts of CD4^+^ and CD8^+^ T lymphocyte subpopulations are important indicators of cellular immune function (Murphy & Reiner, 2002). The increased ratio of CD4^+^/CD8^+^ T lymphocytes caused by AECs suggests that AECs might enhance immune activity. Moreover, our results demonstrated that AECs treatment significantly increased the proportion of CD3^‐^CD161^+^ NK cells and CD3^+^CD161^+^ NKT cells. Interestingly, Kaito Sakaguchi et al observed that the 30–50% ethanol precipitate of oyster extract (EPOE50) enhanced the activity of mouse spleen NK cells in vitro and in vivo and then inhibited tumor growth in a dose‐dependent manner (Sakaguchi et al., 2018). In summary, AECs treatment may regulate the balance of cellular immunity and the body's humoral immunity. However, the underlying molecular mechanisms involved in the changes in the proportion of splenic lymphocyte subsets are currently unknown, and further studies are needed. Activated splenocytes generate and sequentially secrete a variety of inflammatory cytokines to regulate the immune response. An increase in the level of one inflammatory factor can promote the synthesis and secretion of other inflammatory factors through a cascade reaction (Hye & Min, 2016; Jin‐Gyo et al., 2018; Lou et al., 2020). Thus, we detected cytokine levels by ELISA and found that AECs possessed the ability to upregulate the concentrations of TNF‐*α*, IL‐2, IL‐6, IL‐12, and IFN‐γ in splenocytes. This confirmed that AECs were more effective in regulating the cell‐mediated immune response.

Previous studies have indicated that the MAPK family plays a vital role in regulating cell growth, apoptosis, and the response to inflammation or stress and that activated MAPKs regulate the expression of inflammatory cytokine (Kim & Choi, 2015; Kyriakis & Avruch, 1996). In mammalian cells, there are three major MAPK pathways including p38 MAPK, ERK, and JNK (Kim & Choi, 2015). In our study, we detected the activation of p38, ERK, and JNK MAPKs in splenocytes to further investigate the potential mechanism by which AECs promoted the expression of inflammatory cytokines. AECs treatment promoted p38 MAPK phosphorylation, and it is hypothesized that the p38 MAPK pathway contributes to the regulation of AECs‐mediated immunomodulatory activity. As reported, p38 MAPK activation leads to the activation of transcription factor, NF‐κB, which can induce the upregulation of inflammatory cytokine such as TNF‐*α* (Chung et al., 2003; Peng et al., 2003). However, whether there are other pathways such as NF‐κB signaling pathway involved in the regulation of AECs remains to be further studied.

In summary, AECs promote the proliferation of splenocytes, regulate lymphocyte subsets, and alter the expression and secretion of inflammatory cytokines in vitro. However, its precise mechanisms and the effects of AECs in vivo need to be further explored in future studies. Our present results suggested that *C. sikamea* might be developed as an immunostimulant in the future.

## CONFLICTS OF INTEREST

The authors in this study declare that no conflicts of interest exist.
